# All-cause mortality and disease progression in SARS-CoV-2-infected patients with or without antibiotic therapy: an analysis of the LEOSS cohort

**DOI:** 10.1007/s15010-021-01699-2

**Published:** 2021-10-08

**Authors:** Maximilian J. Schons, Amke Caliebe, Christoph D. Spinner, Annika Y. Classen, Lisa Pilgram, Maria M. Ruethrich, Jan Rupp, Susana M. Nunes de Miranda, Christoph Römmele, Janne Vehreschild, Bjoern-Erik Jensen, Maria Vehreschild, Christian Degenhardt, Stefan Borgmann, Martin Hower, Frank Hanses, Martina Haselberger, Anette K. Friedrichs, the LEOSS-study group, Julia Lanznaster, Christoph D. Spinner, Maria Madeleine Ruethrich, Bjoern-Erik Jensen, Martin Hower, Jan Rupp, Christoph Roemmele, Maria Vehreschild, Christian Degenhardt, Stefan Borgmann, Frank Hanses, Kerstin Hellwig, Jürgen vom Dahl, Sebastian Dolff, Christiane Piepel, Jan Kielstein, Silvio Nadalin, Marc Neufang, Milena Milovanovic, Kai Wille, Katja Rothfuss, Lukas Eberwein, Wolfgang Rimili, Timm Westhoff, Maximilian Worm, Gernot Beutel, Norma Jung, Joerg Schubert, Philipp Markart, Jessica Rueddel, Ingo Voigt, Robert Bals, Claudia Raichle, Jörg Janne Vehreschild, Carolin E. M. Jakob, Lisa Pilgram, Melanie Stecher, Maximilian Schons, Susana M. Nunes de Miranda, Nick Schulze, Sandra Fuhrmann, Clara Brünn, Annika Claßen, Bernd Franke, Fabian Praßer, Martin Lablans

**Affiliations:** 1 Department I of Internal Medicine, University Hospital of Cologne, Cologne, Germany; 2 Institute for Medical Informatics and Statistics, University Hospital Schleswig-Holstein, Campus Kiel, Kiel, Germany; 3 Kiel University, Kiel, Germany; 4 School of Medicine, Department of Internal Medicine II, Technical University of Munich, University Hospital Rechts Der Isar, Munich, Germany; 5 German Centre for Infection Research (DZIF), Partner Site Bonn-Cologne, Cologne, Germany; 6 Department II of Internal Medicine, Hematology/Oncology, Goethe University, Frankfurt, Frankfurt am Main, Germany; 7 Institute for Infection Medicine and Hospital Hygiene, University Hospital Jena, Jena, Germany; 8 University Hospital Schleswig-Holstein, Lübeck, Germany; 9 Internal Medicine III – Gastroenterology and Infectious Diseases, University Hospital of Augsburg, Augsburg, Germany; 10 Clinic for Gastroenterology, Hepatology and Infectiology, University Hospital Düsseldorf, Heinrich-Heine-University Düsseldorf, Düsseldorf, Germany; 11 Municipal Hospital Karlsruhe, Karlsruhe, Germany; 12 Department of Infectious Diseases and Infection Control, Ingolstadt Hospital, Ingolstadt, Germany; 13 Department of Pneumology, Infectious Diseases and Intensive Care, Klinikum Dortmund gGmbH, Dortmund, Germany; 14 Interdisciplinary Emergency Department, University Hospital Regensburg, Regensburg, Germany; 15 Department of Internal Medicine I, Passau Hospital, Passau, Germany; 16 Clinic for Internal Medicine I, University Hospital Schleswig-Holstein, Campus Kiel, Kiel, Germany

**Keywords:** COVID-19, Antibiotics, Antibiotic stewardship, Procalcitonin, LEOSS

## Abstract

**Purpose:**

Reported antibiotic use in coronavirus disease 2019 (COVID-19) is far higher than the actual rate of reported bacterial co- and superinfection. A better understanding of antibiotic therapy in COVID-19 is necessary.

**Methods:**

6457 SARS-CoV-2-infected cases, documented from March 18, 2020, until February 16, 2021, in the LEOSS cohort were analyzed. As primary endpoint, the correlation between any antibiotic treatment and all-cause mortality/progression to the next more advanced phase of disease was calculated for adult patients in the complicated phase of disease and procalcitonin (PCT) ≤ 0.5 ng/ml. The analysis took the confounders gender, age, and comorbidities into account.

**Results:**

Three thousand, six hundred twenty-seven cases matched all inclusion criteria for analyses. For the primary endpoint, antibiotic treatment was not correlated with lower all-cause mortality or progression to the next more advanced (critical) phase (*n* = 996) (both *p* > 0.05). For the secondary endpoints, patients in the uncomplicated phase (*n* = 1195), regardless of PCT level, had no lower all-cause mortality and did not progress less to the next more advanced (complicated) phase when treated with antibiotics (*p* > 0.05). Patients in the complicated phase with PCT > 0.5 ng/ml and antibiotic treatment (*n* = 286) had a significantly increased all-cause mortality (*p* = 0.029) but no significantly different probability of progression to the critical phase (*p* > 0.05).

**Conclusion:**

In this cohort, antibiotics in SARS-CoV-2-infected patients were not associated with positive effects on all-cause mortality or disease progression. Additional studies are needed. Advice of local antibiotic stewardship- (ABS-) teams and local educational campaigns should be sought to improve rational antibiotic use in COVID-19 patients.

## Introduction

Coronavirus disease 2019 (COVID-19) resulting from infection with severe acute respiratory syndrome coronavirus type 2 (SARS-CoV-2), first described in Wuhan, China, in late 2019, has become a global pandemic. The role of bacterial superinfections, their influence on the clinical course, and the appropriate use of antibiotics in a primarily viral respiratory disease are becoming increasingly important in this context [1]. In respiratory viral infections such as influenza, bacterial superinfections can lead to higher morbidity and mortality and require timely diagnosis and initiation of antibiotic therapy (ABT) [2]. Publications report bacterial co- and superinfection rates of less than 10% in COVID-19 patients [2, 3], while the percentage of systemic ABT prescribed was over 60% [2]. This discrepancy is also well documented for other viral diseases such as influenza [4, 5], and international and national campaigns on antibiotic stewardship (ABS) intensively address the consequences for hospitals for more than a decade. ABS aims to sustainably reduce the development of antibiotic resistance by creating awareness for rational antibiotic use and optimized antibiotic therapy strategies [6]. The core elements of ABS are reviewing the indication of ABT and optimizing its duration, dosage, and substance selection based on validated clinical criteria and biomarkers [7].

Possible consequences of untreated bacterial co- and superinfections and diagnostic uncertainties confront medical staff with complex decisions regarding ABT initiation, especially in severely affected patients. In a meta-analysis published in 2021, Langfort et al. summarize that there is currently insufficient evidence to support the widespread use of empiric ABT in hospitalized COVID-19 patients [2, 8]. The World Health Organization (WHO) does not recommend initiating ABT for uncomplicated courses of SARS-CoV-2 infection but recommends therapy for moderate to severe courses of illness and clinical suspicion of bacterial co- or superinfection [9, 10]. Especially for patients in the complicated phase of the disease, it is of crucial importance to name contraindications for and effects of ABT on treatment outcomes to provide physicians with decision-making strategies while global COVID-19 case rates stay high [11]. Procalcitonin (PCT) is a validated serological marker for differentiating between bacterial and non-bacterial acute respiratory tract infections. Bacterial infections enhance its production and release from extrathyroidal sources into the circulation and low PCT indicates a lower likelihood for bacterial infection [12–14]. First studies investigated PCT’s relevance for SARS-CoV-2-infected patients [15]. A better understanding of antibiotic therapy guided by (low) PCT in COVID-19, especially for complicated patients, would be beneficial. This study focuses on the association of ABT and the outcomes all-cause mortality and clinical worsening in patients in a complicated phase of COVID-19 and low PCT values.

## Method

### Study design

**Fig. 1:**
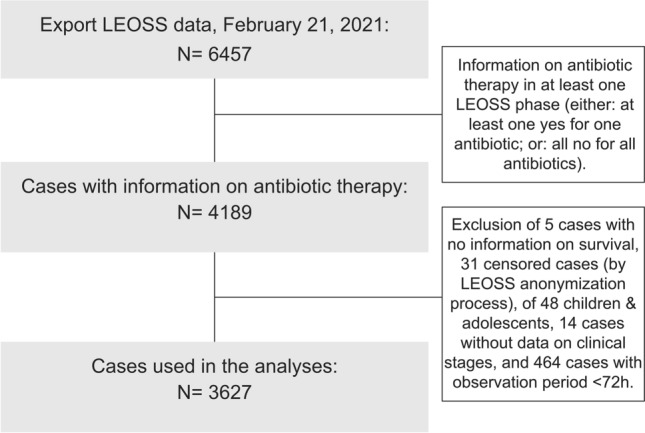
Flowchart showing the inclusion criteria for the analysis. LEOSS: Lean European Open Survey on SARS-CoV-2-Infected Patients

**Fig. 2:**
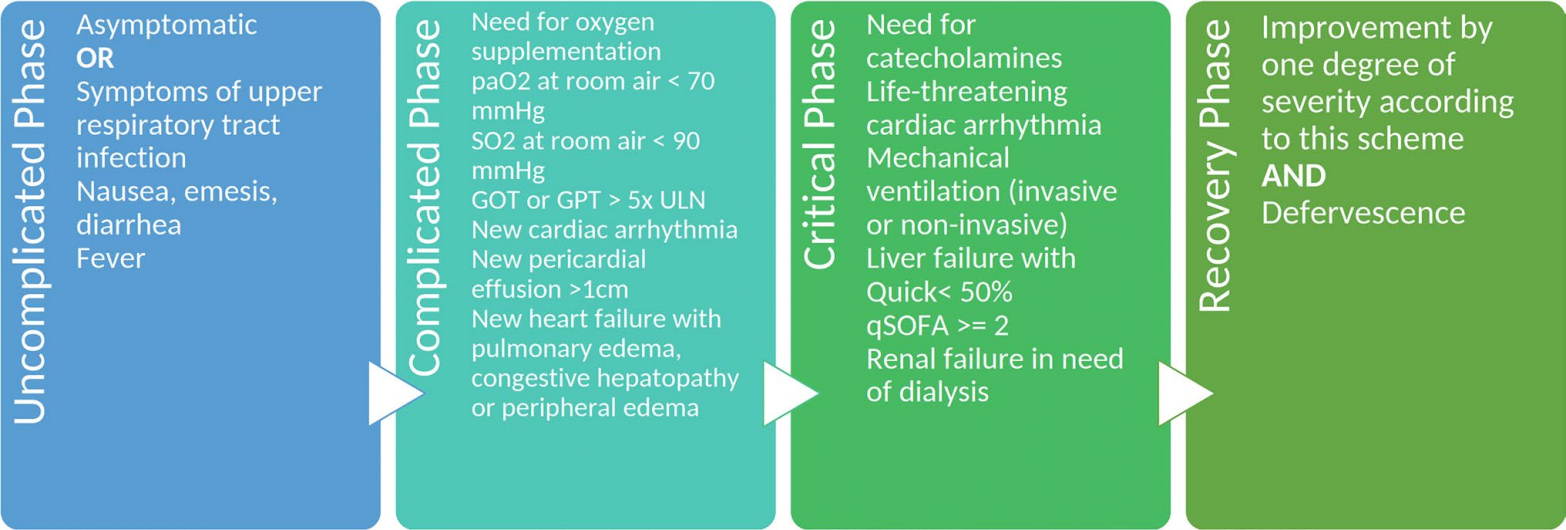
Clinical symptoms and characteristics defining the different phases (uncomplicated [UC], complicated [CO], critical [CR] and recovery) in the LEOSS cohort. The alternative endpoint “death” is not displayed in this figure

This study uses data from the multicenter Lean European Open Survey on SARS-CoV-2-Infected Patients (LEOSS) cohort established in March 2020 (DRKS, No. S00021145, https://www.drks.de/drks_web/navigate.do?navigationId=trial.HTML&TRIAL_ID=DRKS00021145). Cases between March 18, 2020, and February 16, 2021, were included, if they were ≥ 18 years, information on ABT was available, and a minimum observation period of 3 days (≥ 72 h) was reached. In addition to censored cases, those without a documented treatment outcome were excluded (see Fig. 1). PCT was dichotomized by a threshold commonly used for lower respiratory diseases [16, 17] of 0.5 ng/ml (≤ 0.5 ng/ml [PCT↓] and > 0.5 ng/ml [PCT↑]). The clinical outcomes considered in this study were all-cause mortality (yes/no) and progression to the next advanced phase of the disease (yes/no) in the LEOSS schema (see next section and Fig. 2), each until the end of the acute phase of SARS-CoV-2 infection (e.g., recovery, or death).

The primary endpoint of this study was the effect of ABT, defined as any antibiotic agent received irrespective of dose or duration, on all-cause mortality and progression to the critical phase in patients in the complicated phase with low PCT values (PCT↓). Secondary endpoints were the effects of ABT on all-cause mortality and progression to the next advanced phase in patients in the complicated phase with PCT↑ and patients in the uncomplicated phase with PCT↓ and PCT↑.

Possible confounders were chosen after literature review and availability in the data set, resulting in the inclusion of gender, age, and comorbidity state as Charlson comorbidity Index (CCI) [18–24]. We calculated the CCI instead of individual comorbidities to sustain high case numbers for conclusive statistical analysis, [25]. The dataset included binary information on all relevant diseases to calculate the CCI (see also Table 1) [28]. CCI strata of 0–2, 3–4, and > 4 were chosen, reflecting a low, increased, or high comorbidity state, respectively. Where possible, body-mass-index (BMI) and quick Sepsis Related Organ Failure Assessment (qSOFA) were considered. Due to the insufficient data on bacterial superinfections, those were not included in the analysis.

**Table 1:** Baseline characteristics of patients included in the analyses by clinical phase (a) and Influence of risk factors on all-cause mortality and entry into critical (CR)-phase in all COVID-19 patients included in the analysis (b)

a. Baseline characteristics of patients included in the analyses by clinical phase. As patients move through clinical phases, the sum of the phases is higher than the total. Differences of cases to the respective total population in each column account for missing or unknown values				
	Total	Uncomplicated phase	Complicated phase	Critical phase
Included cases	3627	82.6% (2995/3627)	51.0% (1850/3627)	20.2% (731/3627)
Age				
18–25 years	3.0% (111/3627)	3.5% (104/2995)	0.7% (13/1850)	0.5% (9/731)
26–35 years	6.8% (248/3627)	7.9% (237/2995)	2.8% (52/1850)	0.9% (17/731)
36–45 years	8.9% (324/3627)	9.7% (290/2995)	5.9% (109/1850)	2.1% (39/731)
46–55 years	15.8% (574/3627)	16.6% (498/2995)	15.2% (281/1850)	6.1% (113/731)
56–65 years	18.8% (681/3627)	18.4% (552/2995)	18.8% (348/1850)	9.5% (176/731)
66–75 years	17.1% (621/3627)	16.4% (491/2995)	19.2% (355/1850)	9.3% (172/731)
76–85 years	21.1% (766/3627)	19.7% (590/2995)	27.0% (499/1850)	8.8% (163/731)
> 85 years	8.3% (302/3627)	7.8% (233/2995)	10.4% (193/1850)	2.3% (42/731)
Gender				
Male	58,0% (2107/3627)	57.4% (1719/2995)	60.8% (1124/1850)	73.5% (537/731)
Female	42.0% (1520/3627)	42.6% (1276/2995)	39.2% (726/1850)	26.5% (194/731)
Body mass index (kg/m^2^)				
< 18.5	2.2% (52/2293)	2.2% (42/1920)	2.0% (23/1178)	1.4% (7/513)
18.5–24.9	30.4% (697/2293)	32.3% (620/1920)	27.8% (328/1178)	21.4% (110/513)
25–29.9	36.0% (825/2293)	36.3% (696/1920)	34.9% (411/1178)	38.6% (198/513)
30–34.9	19.8% (455/2293)	19.2% (368/1920)	23.0% (271/1178)	21.1% (108/513)
≥ 35	11.5% (264/2293)	10.1% (194/1920)	12.3% (145/1178)	17.5% (90/513)
Comorbidities (as included in the Charlson Comorbidity Index)				
Acute myocardial infarction	5.8% (201/3495)	5.5% (160/2910)	6.6% (117/1767)	6.4% (44/687)
Congestive heart failure	8.8% (307/3493)	8.1% (235/2911)	10.6% (187/1768)	11.7% (80/686)
Peripheral vascular disease	4.4% (154/3491)	4.1% (120/2907)	5.8% (102/1765)	5.4% (37/684)
Cerebral vascular disease	9.1% (320/3509)	8.3% (242/2922)	11.8% (210/1780)	8.6% (59/685)
Dementia	8.9% (313/3501)	8.0% (234/2917)	12.1% (215/1773)	6.6% (45/683)
Pulmonary disease	3.8% (132/3503)	3.4% (98/2916)	4.6% (82/1777)	6.6% (45/681)
Connective tissue disease	0.5% (16/3502)	0.5% (16/2917)	0.3% (5/1776)	0.6% (4/682)
Peptic ulcer disease	1.5% (53/3497)	1.3% (38/2910)	1.6% (28/1772)	2.8% (19/681)
Liver disease	2.0% (69/3503)	1.9% (56/2918)	2.4% (42/1779)	2.7% (19/684)
Diabetes	15.4% (542/3528)	13.8% (403/2927)	18.1% (326/1797)	21.6% (151/698)
Diabetes with complications	7.9% (277/3509)	6.8% (198/2919)	10.1% (181/1784)	11.2% (77/684)
Hemiplegia or paraplegia	1.9% (68/3504)	1.6% (48/2917)	2.4% (42/1775)	2.6% (18/684)
Renal disease	14.9% (524/3519)	13.7% (401/2925)	17.7% (316/1786)	18.9% (130/689)
Cancer (solid tumor)	2.4% (84/3499)	2.3% (66/2914)	2.7% (48/1775)	1.9% (13/681)
Cancer (leukemia)	7.9% (278/3499)	7.9% (229/2916)	9.6% (171/1775)	8.3% (57/685)
Cancer (lymphoma)	1.6% (55/3498)	1.6% (47/2913)	2.0% (36/1776)	2.0% (14/683)
Metastatic cancer	1.0% (36/3500)	1.0% (29/2914)	1.5% (27/1778)	1.3% (9/684)
Severe liver disease	0.9% (31/3501)	1.0% (25/2916)	0.6% (11/1777)	0.9% (6/683)
HIV disease	Censored*	Censored*	Censored*	Censored*

b. Influence of risk factors on all-cause mortality and entry into critical (CR)-phase in all COVID-19 patients included in the analysis (see Methods section for inclusion criteria)						
*n* = 3627	All-cause mortality (death from any cause incl. COVID-19) *n* = 508			Entry into CR-phase *n* = 731		
	*n* (%)	Univariate	Multivariate OR [CI]	*n* (%)	Univariate	Multivariate OR [CI]
Gender						
M (*n* = 2107, 58%)	336 (16%)		Ref	537 (25%)		Ref
F (*n* = 1520, 42%)	172 (11%)		0.6 [0.5–0.7]	194 (13%)		0.43 [0.36–0.52]
NA (*n* = 0)						
* p* value		< 0.001	< 0.001		< 0.001	< 0.001
Age						
18–55 (*n* = 1257, 35%)	38 (3%)		Ref	178 (14%)		Ref
56–75 (*n* = 1302, 36%)	177 (14%)		4.2 [2.9–6.1]	348 (27%)		2.2 [1.8–2.7]
> 75 (*n* = 1068, 29%)	293 (27%)		9.0 [6.3–13.0]	205 (19%)		1.6 [1.2–1.9]
NA (*n* = 0)						
* p* value		< 0.001	< 0.001		< 0.001	< 0.001
Charlson-Comorbidty-Index						
0–2 (*n* = 2826, 78%)	267 (9%)		Ref	547 (19%)		Ref
3–4 (*n* = 471, 13%)	137 (29%)		2.3 [1.8–3.0]	108 (23%)		
> 4 (*n* = 330, 9%)	104 (32%)		2.5 [1.9–3.3]	76 (23%)		
NA (*n* = 0)						
* p* value		< 0.001	< 0.001		0.077	*n*.s
Baseline-qSOFA						
0–1 (*n* = 1590, 92%)	206 (13%)		Too many missing values	292 (18% CI 16.5–20.4)		Too many missing values
2–3 (*n* = 132, 8%)	27 (20%)			36 (27% CI 19.9–35.7)		
NA (*n* = 1905)						
* p* value		0.023			0.015	
Body-Mass-Index						
< 30 (*n* = 1574, 69%)	203 (13%)		Too many missing values	315 (20%)		Too many missing values
> = 30 (*n* = 719, 31%)	96 (13%)			198 (28%)		
NA (*n* = 1334)						
* p* value		0.79			< 0.001	

*m* male, *f* female, age in years, *NA* missing values, *p value p* value for the univariate or multivariate analysis; for the multivariate logistic regression analysis the influence variables gender, age, CCI were included (after backward selection for *p* > 0.05), *n.s.* not significant in multivariate analysis, *CI* 95% confidence interval, *OR* odds ratio, *Ref* reference category, *qSOFA* quick Sequential Organ Failure Assessment

*Censored by LEOSS anonymization pipeline. Read more in the article “Design and evaluation of a data anonymization pipeline to promote Open Science on COVID-19” by Jakob et. al.

### LEOSS cohort

The LEOSS cohort was initiated to identify independent predictors of outcome in patients diagnosed with SARS-CoV-2 and performs no follow-ups. LEOSS collects data from health care records of any outpatients or inpatients with confirmed SARS-CoV-2 infection (either via positive reverse transcriptase-polymerase chain reaction [rtPCR] or rapid antigen test) and completed acute treatment at participating university hospitals, non-university hospitals, and practices [26]. As of March 18, 2021, 133 active study sites with valid ethical votes from 12 European countries are documenting. Study centers outside Germany documented approximately 5% of the cases, the non-university sector approximately 45%. The study protocol excludes pregnant women.

The LEOSS cohort defines three clinical phases of COVID-19 (uncomplicated, complicated, critical) and two outcome phases (recovery and death; see Fig. 2 for details):

- Clinical phases
  o Uncomplicated (UC) phase: oligo-/asymptomatic
  o Complicated (CO) phase: oxygenation or equivalent clinical deterioration
  o Critical (CR) phase: life-sustaining measures
- Outcome phases
  o Recovery: clinical improvement/discharge
  o Death: from COVID-19; from other cause

Depending on the course of the disease, patients moved through multiple phases or skipped up to two clinical phases. Patients can appear in several subgroups, e.g., in both the UC- and the CO-phase. A patient cannot move back to a previous clinical phase.

LEOSS collects an extensive anonymous dataset and provides individual anonymized LEOSS Scientific Use Files (SUFs) for analyses [27]. Anonymization is mainly achieved by summarizing the values of variables into categories. Information on therapy, diagnostics, and interventions is aggregated over each phase. Usually, only one value that deviates the most from the normal range is documented. The electronic case report form (eCRF) enforces binary documentation of therapies and interventions [26]. Due to anonymity and retrospective documentation, inclusion was performed without explicit written consent.

## Statistical analysis

First, all available cases in LEOSS that met the inclusion criteria were characterized by descriptive statistics and analyzed for the influence of the risk factors (age, gender CCI, BMI, and qSOFA) at baseline on clinical outcomes using univariate and multivariate models in an exploratory way. For the primary endpoint, the effect of ABT on clinical outcomes in the CO-phase with PCT↓ was tested in univariate and multivariate models, in the latter case adjusted for age, gender, and CCI in. qSOFA / SOFA and BMI had to be omitted due to too many missing values. A missingness analysis was performed for patients in the CO phase (see Table 5 in the Appendix). It included a comparison of the group of patients with complete information on PCT and ABT against the group of patients with incomplete information on PCT and ABT (i.e., at least one missing value in PCT or ABT) concerning the two clinical outcomes and the risk factors age, gender, CCI, BMI and qSOFA at the time of admission. For the secondary endpoints, patients in the CO-phase with PCT↑ and patients in the UC-phase with PCT↓ or PCT↑ were studied using the same clinical outcomes, influence factors and statistical analyses as for the primary endpoint.

The univariate analyses and the missingness analysis tested the association of individual variables for significance using the Fisher exact test. The multivariate analyses used a logistic regression model. Our model selection used backward selection with a cut-off value of 0.05 for the *p* value. For this purpose, we compared the model with and without the influencing variable under consideration with the *anova* command. We calculated the odds ratios with the 95% confidence interval for the significant influence variables in the multivariate analysis. All analyses used a two-sided significance level of *p* = 0.05. The statistics program R, version 4.0.3. [29], was used for all analyses.

## Results

### Overview

**Fig. 3:**
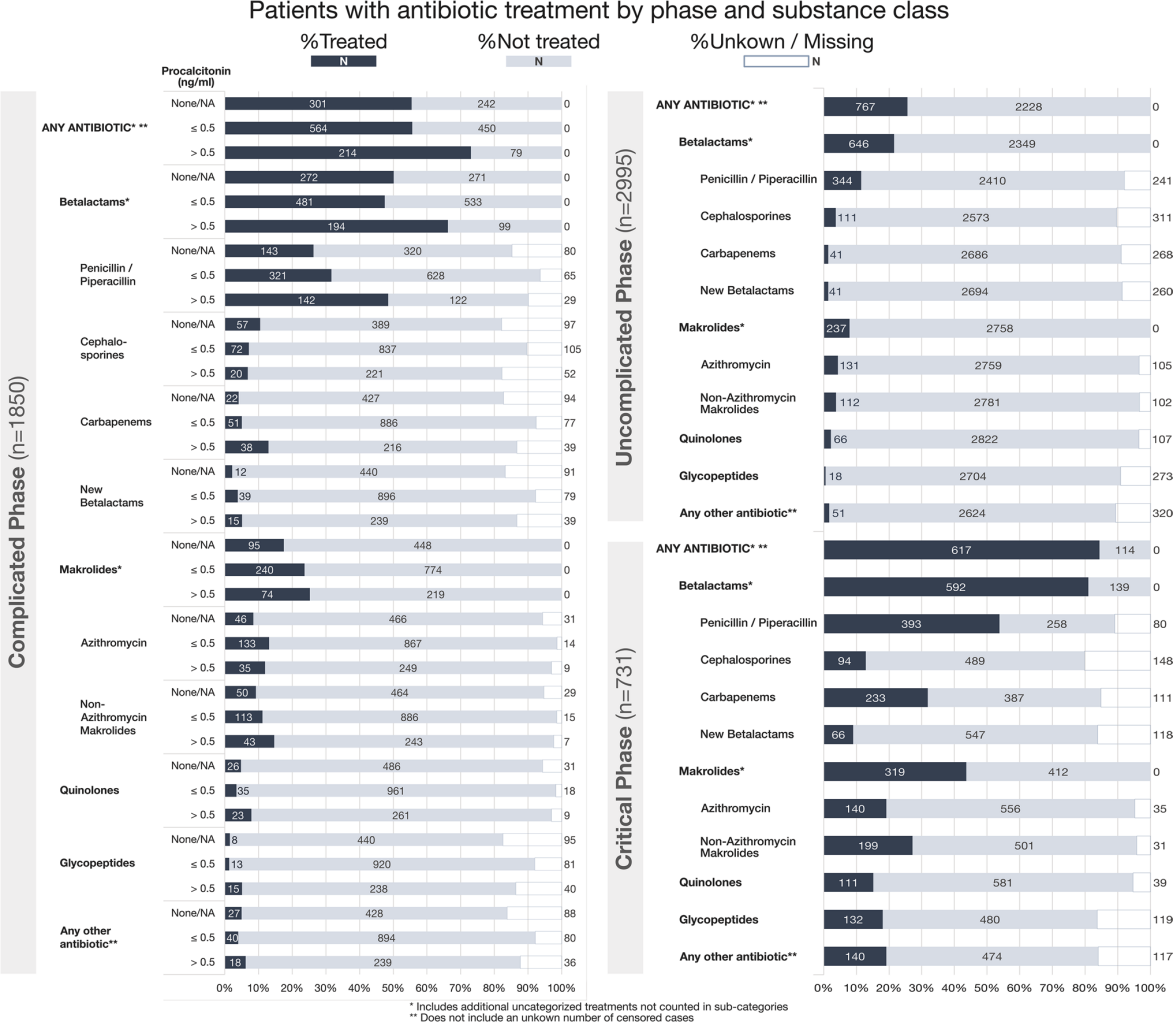
Illustration of antibiotic treatment by clinical phase and antibiotic class. Complicated phase patients are additionally stratified by procalcitonin. Relative percentages are indicated visually by the length of the boxes. Absolute numbers are printed into or next to the respective box. Some antibiotic groups include additional cases not counted in the subgroups due to the documentation process (e.g., “Betalactams”, includes “Penicillin/ Piperacillin”, “Cephalosporins”, “Carbapenems”. “New betalactams” and Betalactams where the exact type is not specified). A patient can both show up in multiple phases (disease progression) and in multiple antibiotic classes (e.g., multiple antibiotic classes used for treatment), but will be counted once only for a given antibiotic class in a phase (e.g., multiple Betalactam treatments of the same subgroup will be counted as one)

Of all patients documented in the LEOSS registry at the time of our analysis *n* = 6457 data on antibiotic use in at least one clinical phase were available in 4189 cases. Five cases had to be removed due to lack of information on survival and another 31 cases due to censored variables, 48 cases due to age < 18 years, 14 cases due to missing data on clinical stages, and 464 cases due to an observation period < 72 h. The results below refer to the remaining 3627 cases (see Fig. 1). Of those 3627 patients, 1024 had missing information on PCT across all phases. Table 1 a summarizes the patient characteristics of the cohort. In Fig. 3, relative and absolute ABT is illustrated for both clinical stage and antibiotic class—for CO-phase patients additionally broken down by PCT levels. In the UC-phase 25.6% (767/2995) received any ABT, 58.3% (1079/1850) and 84.4% (617/731) in the CO-phase and CR-phase, respectively.

Of the 3627 patients included, 508 (14.0%) died. Seven hundred thirty-one (20.2%) reached the CR-phase. In the multivariate analysis of the total study population, male gender (female: OR 0.6 [0.5–0.7], *p* < 0.001; reference male), advanced age (age 56–75: OR 4.2 [2.9–6.1]; age > 75: OR 9.0 [6.3–13.0], *p* < 0.001; reference age 18–55) and a high CCI (CCI 3–4: OR 2.3 [1.8–3.0]; CCI > 4: OR 2.5 [1.9–3.3], *p* < 0.001; reference CCI 0–2) were significantly associated with a higher all-cause mortality (see Table 1 b). In particular, age > 75 compared to the reference group (18–55 years) showed significantly increased death rates. For entering the CR-phase, cases with male gender (female: OR 0.43 [0.36–0.52], *p* < 0.001; reference male) and higher age (age 56–75: OR 2.2 [1.8–2.7]; age > 75: OR 1.6 [1.2–1.9], *p* < 0.001; reference age 18–55) showed a significantly increased probability. The CCI did not correlate with a higher chance of entering the CR-phase. In the univariate analysis, a baseline qSOFA of > 1 also showed a significantly higher risk of death (*p* = 0.023) but no significant association for entering the CR-phase. A BMI of > 30, on the other hand, was a significant risk factor only for entry into the CR-phase (*p* < 0.001) and not for higher all-cause mortality. With too many missing values, we did not include BMI and qSOFA in the multivariate analyses.

### Antibiotic treatment in the clinical phases stratified by PCT levels

#### Primary endpoint: antibiotic therapy in CO-phase patients with PCT levels ≤ 0.5 ng/ml

60.4% (602/996) of CO-phase PCT↓ patients received ABT, 39.6% (394/996) did not. In the multivariate analysis, ABT had no significant association with all-cause mortality or entry into the next more advanced stage (CR-phase) (*p* > 0.05) when adjusting for the possible confounders gender, age, and CCI (see Table 2). Female gender was significantly associated with lower all-cause mortality (OR 0.7 [0.4–1.0], *p* = 0.039; reference male) and less frequent entry into the CR-phase (OR 0.5 [0.4–0.7], *p* < 0.001, reference male). Higher age showed a significant association with a strong increase in all-cause mortality (age 56–75: OR 4.6 [1.8–12.1]; age > 75: OR 13.0 [5.1–33.4], *p* < 0.001; reference age 18–55); the same applies to an increased CCI (CCI 3–4: OR 2.5 [1.6–3.9]; CCI > 4: OR 2.7 [1.5–4.6], *p* < 0.001; reference CCI 0–2). Age and CCI were not associated with an increased probability of entering the CR-phase (*p* > 0.05).

**Table 2:** Influence of risk factors on all-cause mortality and progression into the next more advanced stage (critical [CR]-phase) in COVID-19 patients in the complicated (CO)-phase with procalcitonin levels ≤ 0.5 ng/ml (PCT↓)

*n* = 996	All-cause mortality (death from any cause incl. COVID-19) *n* = 138			Progression into next more advanced stage (CR-phase) *n* = 185		
	*n* (%)	Univariate	Multivariate OR [CI]	*n* (%)	Univariate	Multivariate OR [CI]
Antibiotic treatment						
Yes (*n* = 602, 60%)	99 (16%)		Ref	121 (20%)		Ref
No (*n* = 394, 40%)	39 (10%)			64 (16%)		
NA (*n* = 0)						
* p* value		0.0036	n.s		0.13	n.s
Gender						
M (*n* = 588, 59%)	85 (14%)		Ref	131 (22%)		Ref
F (*n* = 408, 41%)	53 (13%)		0.7 [0.4–1.0]	54 (13%)		0.5 [0.4–0.7]
NA (*n* = 0)						
* p* value		0.58	0.039		< 0.001	< 0.001
Age						
18–55 (*n* = 259, 26%)	5 (2%)		Ref	45 (17%)		Ref
56–75 (*n* = 373, 37%)	37 (10%)		4.6 [1.8–12.1]	78 (21%)		
> 75 (*n* = 364, 37%)	96 (26%)		13.0 [5.1–33.4]	62 (17%)		
NA (*n* = 0)						
* p* value		< 0.001	0.001		0.35	n.s
Charlson Comorbidity Index						
0–2 (*n* = 768, 77%)	69 (9%)		Ref	133 (17%)		Ref
3–4 (*n* = 144, 14%)	42 (29%)		2.5 [1.6–3.9]	34 (24%)		
> 4 (*n* = 84, 8%)	27 (32%)		2.7 [1.5–4.6]	18 (21%)		
NA (*n* = 0)						
* P* value		< 0.001	< 0.001		0.15	n.s

*m* male, *f* female, age in years, *NA* missing values, *p value p* value for the univariate or multivariate analysis; for the multivariate logistic regression analysis the influence variables gender, age, CCI were included (after backward selection for *p* > 0.05), *n.s.* not significant in the multivariate analysis, *CI* 95% confidence interval, *OR* odds ratio, *Ref* reference category

65% of administered antibiotics in this patient population were Betalactam antibiotics; approximately a quarter of the cases received Macrolides; Quinolones were used in 4% of the cases (data not shown). The missingness analysis of patients in the CO-phase showed significant differences between patients with and without missing data of ABT and PCT with respect to progression into the CR-phase (*p* < 0.001) but not for age, gender, ABT, CCI strata, BMI, and baseline qSOFA (*p* > 0.05, see Table 6 in the Appendix).

#### Secondary analyses: Antibiotic therapy in other clinical constellations

In the UC-phase PCT↓ subgroup, 38.1% (399/1045) of patients received ABT, and 61.9% (646/1045) did not. Here, age and CCI (all *p* < 0.001), but not ABT and gender (both *p* > 0.05), were significantly associated with increased all-cause mortality in the multivariate analysis (see Table 3 in the Appendix). The entry of this subgroup into the CO-phase was significantly associated with ABT and age (both *p* < 0.001), but not with an increased CCI (*p* > 0.05).

In the UC-phase PCT↑, 69.3% (104/150) of patients received systemic antibiotic therapy, and 30.7% (46/150) did not (see Table 4 in the Appendix). All-cause mortality and entry into the CO-phase were increased for age (*p* < 0.001, *p* = 0.020, respectively). Analyses for gender, ABT, and CCI strata yielded no significant associations for either all-cause mortality or entry in the CO-phase (*p* > 0.05).

In the CO-phase PCT↑ subgroup, antibiotics were prescribed in 85.3% (244/286) of patients and not prescribed in 14.7% (42/286) (see Table 5 in the Appendix). Patients with ABT or age > 55 years had a significantly increased risk of death from any cause (*p* = 0.029, *p* < 0.001, respectively). Male gender was the only parameter that showed a statistically significant difference in this subgroup for entry into the CR-phase (*p* = 0.0034).

## Discussion

In this cohort of SARS-CoV-2-infected patients with documented information on ABT, established risk factors such as male gender, patient age > 55 years, and CCI ≥ 3 were significantly associated with all-cause mortality. Similar results have been reported before [18–24]. For the primary endpoint, CO-phase patients with PCT↓, no significant correlation between antibiotic treatment and all-cause mortality or progression to the critical phase was seen.

This study's additional subgroup analyses found similar results, in line with WHO’s recommendations [10]. For neither the primary nor the secondary endpoints a significant benefit of ABT could be demonstrated. CO-phase PCT↑ patients with ABT had increased all-cause mortality, UC-phase PCT↓ patients with ABT had a higher likelihood to enter the complicated phase. For both, we highly suspect a worsening clinical course to trigger ABT, with the former being the driving factor for increased mortality/progression and the latter being an intervention of uncertain benefit or harm. The clinical state of the patient probably is a classical confounder. Unfortunately, in our cohort we do not have a clinical severity score (e.g., SOFA) available.

Surprisingly, the CCI was no significant risk factor for progression into a more advanced phase in any of the analyses, but was associated with all-cause mortality in both UC- and CO-phase patients with PCT↓. Palliative care concepts for multimorbid patients could be a possible explanatory hypothesis here. Difficulties with this outcome are also reflected by a significant difference between patients in the missingness analysis for the complicated phase.

SARS-CoV-2 infections are frequently co-treated with antibiotics in the LEOSS cohort, regardless of the respective phase. International publications report similarly high rates of antibiotic prescriptions [30, 31]. The antibiotics administered mainly matched the empirical antibiotics recommended in guidelines for community-acquired or nosocomial pneumonia [32, 33]. The proportion of antibiotics with *Pseudomonas*- or *Methicillin-resistant Staphylococcus aureus* (MRSA) activity was comparatively low compared to a study from South Korea [34].

## Strength of this study

To the best of our knowledge, our analysis is the largest evaluation of antibiotic therapy effects on mortality and disease progression in a German SARS-CoV-2-infected population. Data collection took place at > 100 recruiting sites with an intersectoral recruitment approach across university hospitals, non-university hospitals, and primary care practices. Anonymous recruitment allowed for broad inclusion of patients reducing selection bias [35]. The study population’s characteristics seem to be representative of German [18, 36] and international cohorts of hospitalized COVID-19 patients [19–24, 30]. The study population includes cases from the first and second waves of the COVID-19 pandemic in Germany. Our analysis includes established risk factors and is stratified by typical PCT thresholds for lower respiratory infections [16, 17]. We stratified patients according to their clinical phase to obtain more robust results.

## Limitations

As a retrospective, non-randomized analysis, some limitations need to be considered when assessing our results. The analyzed patient population did not include pregnant women and individuals < 18 years. We excluded pediatric cases due to low case numbers and the broad heterogeneity of this patient collective ranging from neonate to young adult. Our data did not provide reasons for the initiation of ABT, and high-quality superinfection data was not available. Our analyses thus assume the administration of antibiotics in the context of COVID-19 (co-)therapy and suspected bacterial superinfection. However, reasons for antibiotic therapy could often be independent of a SARS-CoV-2 infection, e.g., typical infections such as urinary tract infections or catheter-associated infections [37, 38]. The LEOSS cohort potentially contains numerous patients who were not primarily hospitalized because of COVID-19 but instead had a SARS-CoV-2 infection as a secondary diagnosis (e.g., asymptomatic coinfection or nosocomial infection).

LEOSS’ study design introduces further limitations. First, LEOSS has no dedicated review process of the data beyond automated plausibility checks and queries for implausible cases. Second, there is no follow-up after the acute course. Hence, we could not include higher re-hospitalization rates or post-discharge effects in our analyses and endpoints are limited to the end of the acute infection (e.g., until discharge or recovery). Third, the analysis could not include essential information about repetition, course, period, and dosage of antibiotic therapies or microbiological or radiological diagnostics and the relationship between events within a phase due to anonymous data acquisition. For example, early discontinuation of an antibiotic prescription that low PCT levels might trigger cannot be observed in our dataset and thus is not accounted for in the analyses.

Finally, although our analysis considers many covariates, additional risk factors are described in the literature, e.g., socioeconomic or genetic factors [39, 40], that were not taken into account. These variables were either not present or insufficiently documented as for the BMI or SOFA scores. Given that the clinical presentation is probably the essential factor for physicians’ initial assessment for or against ABT, the lack of a marker for clinical presentation, such as the SOFA, is probably the most substantial limitation of our analysis.

## Conclusion

In summary, the data and analyses of ABT in SARS-CoV-2-infected patients presented here do not demonstrate a correlation of ABT with lower all-cause mortality or protection from progression to the next more advanced phase of disease for uncomplicated or complicated patients irrespective of PCT levels. The limitations of our available cohort data demand further comprehensive studies, such as the German National Pandemic Cohort Network (NAPKON). Antibiotic-resistant bacteria are another severe and global pandemic and many authors already called for conscious ABS activities in times of COVID-19 [1, 4, 34, 41, 42]. The involvement of local ABS-teams or ABS-commissioned physicians in the decision process for or against antibiotic therapy in COVID-19 patients, in addition to educational campaigns focused on rational use of antibiotics, remains of crucial importance [41].

## Data Availability

A scientific use file (SUF) can be requested from the LEOSS analysis team via www.leoss.net.

## Appendix

**Table 3:** Influence of risk factors on all-cause mortality and progression into the next more advanced stage (complicated [CO]-phase) in COVID-19 patients in the uncomplicated (UC)-phase with procalcitonin levels ≤ 0.5 ng/ml (PCT↓)

*n* = 1045	All-cause mortality (death from any cause incl. COVID-19) *n* = 50	Progression into next more advanced stage (CO-phase) *n* = 287
Antibiotic treatment		
Yes (*n* = 399, 38%)	25 (6% CI 4.1–9.1)	136 (34% CI 29.4–39.0)
No (*n* = 646, 62%)	25 (4% CI 2.5–5.7)	151 (23% CI 20.2–26.8)
*p* (*p*-mult)	0.10 (*n*.s.)	< 0.001 (< 0.001)
Gender		
M (*n* = 565, 54%)	28 (5% CI 3.3–7.1)	158 (28% CI 24.3–31.9)
F (*n* = 480, 46%)	22 (5% CI 2.9–6.9)	129 (27% CI 23.0–31.1)
*p* (*p*-mult)	0.88 (n.s.)	0.73 (n.s.)
Age		
18–55 (*n* = 412, 39%)	1 (0.2% CI 0.006–1.3)	70 (17% CI 13.5–21.0)
56–75 (*n* = 361, 35%)	15 (4% CI 2.3–6.8)	104 (29% CI 24.2–33.8)
> 75 (*n* = 272, 26%)	34 (13% CI 8.8–17.0)	113 (42% CI 35.6–47.7)
*p* (*p*-mult)	< 0.001 (< 0.001)	< 0.001 (< 0.001)
Charlson Comorbidity Index		
0–2 (*n* = 851, 81%)	20 (2% CI 1.4–3.6)	213 (25% CI 22.2–28.1)
3–4 (*n* = 107, 10%)	17 (16% CI 9.5–24.2)	47 (44% CI 34.3–53.9)
> 4 (*n* = 87, 8%)	13 (15% CI 8.2–24.2)	27 (31% CI 21.5–41.9)
*p* (*p*-mult)	< 0.001 (< 0.001)	< 0.001 (n.s.)

*m* male, *f* female, age in years, *p p* value of the univariate analysis, *p-mult p* value for the multivariate logistic regression analysis with the multiple influence variables antibiotics, gender, age, CCI (after backward selection for *p* > 0.05), *n.s.* not significant in the multivariate analysis, *CI* 95% confidence interval

**Table 4:** Influence of risk factors on all-cause mortality and progression into the next more advanced stage (complicated [CO]-phase) in COVID-19 patients in the uncomplicated (UC)-phase with procalcitonin levels > 0.5 ng/ml (PCT↑)

*n* = 150	All-cause mortality (death from any cause incl. COVID-19) *n* = 25	Progression into next more advanced stage (CO-phase) *n* = 55
Antibiotic treatment		
Yes (*n* = 104, 69%)	20 (19% CI 12.2–28.1)	42 (40% CI 30.9–50.5)
No (*n* = 46, 31%)	5 (11% CI 3.6–23.6)	13 (28% CI 16.0–43.5)
*p* (*p*-mult)	0.24 (n.s.)	0.20 (n.s.)
Gender		
M (*n* = 95, 63%)	18 (19% CI 11.6–28.3)	37 (39% CI 29.1–49.5)
F (*n* = 55, 37%)	7 (13% CI 5.3–24.5)	18 (33% CI 20.7–46.7)
*p* (*p*-mult)	0.37 (n.s.)	0.49 (n.s.)
Age		
18–55 (*n* = 52, 35%)	3 (6% CI 1.2–15.9)	12 (23% CI 12.5–36.8)
56–75 (*n* = 61, 41%)	7 (11% CI 4.7–22.2)	24 (39% CI 27.1–52.7)
> 75 (*n* = 37, 25%)	15 (41% CI 24.8–57.9)	19 (51% CI 34.4–68.1)
*p* (*p*-mult)	< 0.001 (< 0.001)	0.020 (0.020)
Charlson Comorbidity Index		
0–2 (*n* = 94, 63%)	12 (13% CI 6.8–21.2)	34 (36% CI 26.5–46.7)
3–4 (*n* = 30, 20%)	6 (20% CI 7.7–28.6)	12 (40% CI 22.7–59.4)
> 4 (*n* = 26, 17%)	7 (27% CI 11.6–47.8)	9 (35% CI 17.2–55.7)
*p* (*p*-mult)	0.19 (n.s.)	0.91 (n.s.)

*m* male, *f* female, age in years, *p p* value of the univariate analysis, *p-mult* *p* value for the multivariate logistic regression analysis with the multiple influence variables antibiotics, gender, age, CCI (after backward selection for *p* > 0.05), *n.s.* not significant in the multivariate analysis, *CI* 95% confidence interval

**Table 5:** Influence of risk factors on all-cause mortality and progression into the next more advanced stage (critical [CR]-phase) in COVID-19 patients in the complicated (CO)-phase with procalcitonin levels > 0.5 ng/ml (PCT↑)

*n* = 286	All-cause mortality (death from any cause incl. COVID-19) *n* = 109	Progression into next more advanced stage (CR-phase) *n* = 83
Antibiotic treatment		
Yes (*n* = 244, 85%)	98 (40% CI 34.0–46.7)	70 (29% CI 23.1–34.8)
No (*n* = 42, 15%)	11 (26% CI 13.9–42.0)	13 (31% CI 17.6–47.1)
*p* (*p*-mult)	0.089 (0.029)	0.85 (n.s.)
Gender		
M (*n* = 195, 68%)	75 (38% CI 31.6–46.1)	67 (34% CI 27.7–41.5)
F (*n* = 91, 32%)	34 (37% CI 27.4–48.1)	16 (18% CI 10.4–27.0)
*p* (*p*-mult)	0.90 (n.s.)	0.0034 (0.0034)
Age		
18–55 (*n* = 50, 17%)	7 (14% CI 5.8–26.7)	17 (34% CI 21.2–48.8)
56–75 (*n* = 104, 36%)	26 (25% CI 17.0–34.4)	35 (34% CI 24.7–43.6)
> 75 (*n* = 132, 46%)	76 (58% CI 48.7–66.1)	31 (23% CI 16.5–31.6)
*p* (*p*-mult)	< 0.001 (< 0.001)	0.16 (n.s.)
Charlson Comorbidity Score		
0–2 (*n* = 166, 58%)	49 (30% CI 22.7–37.1)	55 (33% CI 26.0–40.8)
3–4 (*n* = 65, 23%)	31 (48% CI 35.1–60.5)	14 (22% CI 12.3–33.5)
> 4 (*n* = 55, 19%)	29 (53% CI 38.8–66.3)	14 (25% CI 14.7–39.0)
*p* (*p*-mult)	0.0017 (n.s.)	0.19 (n.s.)

*m* male, *f* female, age given in years, *p value p* value of the univariate analysis, *p-mult p* value for the multivariate logistic regression analysis with the multiple influence variables antibiotics, gender, age, CCI (after backward selection for *p* > 0.05), *n.s.* not significant in the multivariate analysis, *CI* 95% confidence interval

**Table 6:** Missing analysis for patients in the complicated (CO)-phase comparing complete and incomplete information on antibiotic treatment (ABT) and procalcitonin (PCT)

	ABT and PCT in CO available *n* = 1282	ABT or PCT in CO not available *n* = 703	*p* value comparison complete vs. missing data
Death from any cause yes / no (% death)	247/1035 (19% CI 17.1–21.5)	138/565 (20% CI 16.8–22.8)	0.86
Entry into CR-phase yes / no (% entry)	268/1014 (21% CI 18.7–23.2)	200/503 (28% CI 25.1–31.9)	< 0.001
Gender: m/f (% male)	783/499 (61% CI 58.3–63.8)	426/277 (61% CI 56.9–64.2)	0.85
Age groups (%)	18–55: 309 (24% CI 21.8–26.5) 56–75: 477 (37% CI 34.6–39.9) > 75: 496 (39% CI 36.0–41.4)	18–55: 176 (25% CI 21.9–28.4) 56–75: 287 (41% CI 37.2–44.6) > 75: 240 (34% CI 30.6–37.8)	0.12
CCI groups (%)	0–2: 934 (73% CI 70.3–75.3) 3–4: 209 (16% CI 14.3–18.4) > 4: 139 (11% CI 9.2–12.7)	0–2: 515 (73% CI 69.8–76.5) 3–4: 116 (17% CI 13.8–19.5) > 4: 72 (10% CI 8.1–12.7)	0.92
qSOFA Baseline (%)	0–1: 606 (91% CI 88.3–92.8) 2–3: 62 (9% CI 7.2–11.7) NA: 614	0–1: 308 (91% CI 87.3–93.7) 2–3: 31 (9% CI 6.3–12.7) NA: 364	0.25
BMI Baseline (%)	< 30: 523 (64% CI 61.0–67.7) > = 30: 289 (36% CI 32.3–39.0) NA: 470	< 30: 280 (66% CI 61.2–70.4) > = 30: 145 (34% CI 29.6–38.8) NA: 278	0.40

*m* male, *f* female, age in years, *NA* missing values, *CI* 95% -confidence interval

## Code availability

Upon reasonable request from corresponding author.
