# Correction to: All-cause mortality and disease progression in SARS-CoV-2-infected patients with or without antibiotic therapy: an analysis of the LEOSS cohort

**DOI:** 10.1007/s15010-021-01727-1

**Published:** 2021-12-15

**Authors:** Maximilian J. Schons, Amke Caliebe, Christoph D. Spinner, Annika Y. Classen, Lisa Pilgram, Maria M. Ruethrich, Jan Rupp, Susana M. Nunes de Miranda, Christoph Römmele, Janne Vehreschild, Bjoern-Erik Jensen, Maria Vehreschild, Christian Degenhardt, Stefan Borgmann, Martin Hower, Frank Hanses, Martina Haselberger, Anette K. Friedrichs, the LEOSS-study group, Julia Lanznaster, Christoph D. Spinner, Maria Madeleine Ruethrich, Bjoern-Erik Jensen, Martin Hower, Jan Rupp, Christoph Roemmele, Maria Vehreschild, Christian Degenhardt, Stefan Borgmann, Frank Hanses, Kerstin Hellwig, Jürgen vom Dahl, Sebastian Dolff, Christiane Piepel, Jan Kielstein, Silvio Nadalin, Marc Neufang, Milena Milovanovic, Kai Wille, Katja Rothfuss, Lukas Eberwein, Wolfgang Rimili, Timm Westhoff, Maximilian Worm, Gernot Beutel, Norma Jung, Joerg Schubert, Philipp Markart, Jessica Rueddel, Ingo Voigt, Robert Bals, Claudia Raichle, Jörg Janne Vehreschild, Carolin E. M. Jakob, Lisa Pilgram, Melanie Stecher, Maximilian Schons, Susana M. Nunes de Miranda, Nick Schulze, Sandra Fuhrmann, Clara Brünn, Annika Claßen, Bernd Franke, Fabian Praßer, Martin Lablans

**Affiliations:** 1 Department I of Internal Medicine, University Hospital of Cologne, Cologne, Germany; 2 Institute for Medical Informatics and Statistics, University Hospital Schleswig-Holstein, Campus Kiel, Kiel, Germany; 3 Kiel University, Kiel, Germany; 4 School of Medicine, Department of Internal Medicine II, Technical University of Munich, University Hospital Rechts Der Isar, Munich, Germany; 5 German Centre for Infection Research (DZIF), Partner Site Bonn-Cologne, Cologne, Germany; 6 Department II of Internal Medicine, Hematology/Oncology, Goethe University, Frankfurt, Frankfurt am Main, Germany; 7 Institute for Infection Medicine and Hospital Hygiene, University Hospital Jena, Jena, Germany; 8 University Hospital Schleswig-Holstein, Lübeck, Germany; 9 Internal Medicine III – Gastroenterology and Infectious Diseases, University Hospital of Augsburg, Augsburg, Germany; 10 Clinic for Gastroenterology, Hepatology and Infectiology, University Hospital Düsseldorf, Heinrich-Heine-University Düsseldorf, Düsseldorf, Germany; 11 Municipal Hospital Karlsruhe, Karlsruhe, Germany; 12 Department of Infectious Diseases and Infection Control, Ingolstadt Hospital, Ingolstadt, Germany; 13 Department of Pneumology, Infectious Diseases and Intensive Care, Klinikum Dortmund gGmbH, Dortmund, Germany; 14 Interdisciplinary Emergency Department, University Hospital Regensburg, Regensburg, Germany; 15 Department of Internal Medicine I, Passau Hospital, Passau, Germany; 16 Clinic for Internal Medicine I, University Hospital Schleswig-Holstein, Campus Kiel, Kiel, Germany

## Correction to: Infection 10.1007/s15010-021-01699-2

The original version of this article unfortunately contained a mistake.

In this article the author name Susana M. Nunes de Miranda was incorrectly written as Susana Nunes de Miranda.

The original article has been corrected.

